# Molecular characterization and antiapoptotic function analysis of the duck plague virus Us5 gene

**DOI:** 10.1038/s41598-019-41311-0

**Published:** 2019-03-19

**Authors:** Chuankuo Zhao, Tianqiong He, Yang Xu, Mingshu Wang, Anchun Cheng, XinXin Zhao, Dekang Zhu, Shun Chen, Mafeng Liu, Qiao Yang, Renyong Jia, Xiaoyue Chen, Ying wu, Shaqiu Zhang, Yunya Liu, Yanling Yu, Ling Zhang

**Affiliations:** 1 Institute of Preventive Veterinary Medicine, Sichuan Agricultural University, Wenjiang, Chengdu City, Sichuan 611130 People’s Republic of China; 2 Key Laboratory of Animal Disease and Human Health of Sichuan Province, Sichuan Agricultural University, Wenjiang, Chengdu City, Sichuan 611130 People’s Republic of China; 3 Research Center of Avian Disease, College of Veterinary Medicine, Sichuan Agricultural University, Wenjiang, Chengdu City, Sichuan 611130 People’s Republic of China

## Abstract

Thus far, there have been no reports on the molecular characterization and antiapoptotic function of the DPV Us5 gene. To perform molecular characterization of DPV Us5, RT-PCR and pharmacological inhibition tests were used to ascertain the kinetic class of the Us5 gene. Western blotting and an indirect immunofluorescence assay (IFA) were used to analyze the expression level and subcellular localization of Us5 in infected cells at different time points. Us5 in purified DPV virions was identified by mass spectrometry. The results of RT-PCR, Western blotting, and pharmacological inhibition tests revealed that Us5 is transcribed mainly in the late stage of viral replication. The IFA results revealed that Us5 was localized throughout DPV-infected cells but was localized only to the cytoplasm of transfected cells. Mass spectrometry and Western blot analysis showed that Us5 was a virion component. Next, to study the antiapoptotic function of DPV Us5, we found that DPV CHv without gJ could induce more apoptosis cells than DPV-CHv BAC and rescue virus. we constructed a model of apoptosis in duck embryo fibroblasts (DEFs) induced by hydrogen peroxide (H_2_O_2_). Transfected cells expressing the Us5 gene were protected from apoptosis induced by H_2_O_2_, as measured by a TUNEL assay, a caspase activation assay and Flow Cytometry assay. The TUNEL assay and Flow Cytometry assay results showed that the recombinant plasmid pCAGGS-Us5 could inhibit apoptosis induced by H_2_O_2_ in DEF cells. However, caspase-3/7 and caspase-9 protein activity upregulated by H_2_O_2_ was significantly reduced in cells expressing the recombinant plasmid pCAGGS-Us5. Overall, these results show that the DPV Us5 gene is a late gene and that the Us5 protein is a component of the virion, is localized in the cytoplasm, and can inhibit apoptosis induced by H_2_O_2_ in DEF cells.

## Introduction

Duck plague caused by the duck plague virus (DPV) is an acute hemorrhagic disease that results in sizable economic losses in the avian industry worldwide due to the low egg laying rates and high mortality rates of infected ducks^1–7^.

DPV, a member of the alphaherpesvirus subfamily, has a genome consisting of linear double-stranded DNA comprising a unique long (UL) region, a unique short (US) region, a unique short internal repeat (IRS) region, and a unique short terminal repeat (TRS) region^2,3,8^. The genomic arrangement pattern is UL-IRS-US-TRS. The Us5 gene is highly nonconserved in alphaherpesviruses, and Us5 genes have been described in other Alphaherpesvirinae subfamily members, including herpes simplex virus 1 (HSV-1)^9^, equine herpesvirus-1 (EHV-1)^10,11^, infectious laryngotracheitis virus (ILTV)^12^, and varicella-zoster virus (VZV)^13^. The Us5 protein does not play a role in virus replication and infection like most glycoproteins, but it can regulate the release of the subvirus^14,15^. Many gene products of alphaherpesviruses, including Us5, have antiapoptotic functions^16–20^. The Us5 protein of HSV-1 inhibits apoptosis caused by Fas, UV and granzyme B^18^. The regions of Us5 that inhibit apoptosis are the signal sequence, the extracellular domain and the transmembrane domain^21^, but the antiapoptotic mechanism of Us5 is not clear. Based on the past experimental results, we just know that Us5 protein can regulate caspases, cause mitochondrial membrane potential decline, and promote the production of reactive oxygen species (ROS)^18,21^.

Apoptosis is an important mechanism of host immune defense. The apoptotic process is mainly characterized by cell shrinkage, chromatin aggregation, and apoptotic body formation. Thus far, apoptosis has been shown to be induced by two classical pathways: the extrinsic and intrinsic apoptotic pathways. Caspases are cysteine proteases that are extremely important for intracellular apoptotic pathways, and caspase-9 and caspase-8 are involved in the intrinsic and extrinsic apoptotic pathways, respectively; both caspase-8 and caspase-9 activate the downstream molecule caspase-3 to initiate apoptosis. H_2_O_2_ is an apoptosis inducer that causes cells to undergo oxidative stress and increases intracellular ROS. ROS reduce the mitochondrial membrane potential and activate caspase-9, which subsequently activates the downstream molecule caspase-3^22^.

Our laboratory has previously demonstrated that the function of DPV Us5 is slightly impaired in viral replication, virion assembly and cell-to-cell spread and that is not essential in virion envelopment^23^. However, information regarding the DPV Us5 gene is limited. In this study, we further performed a molecular characterization and investigated the antiapoptotic function of the DPV Us5 gene. The results of this study will provide a foundation for studying the pathogenesis of DPV.

## Results

### Kinetics of DPV Us5

**Figure 1:**
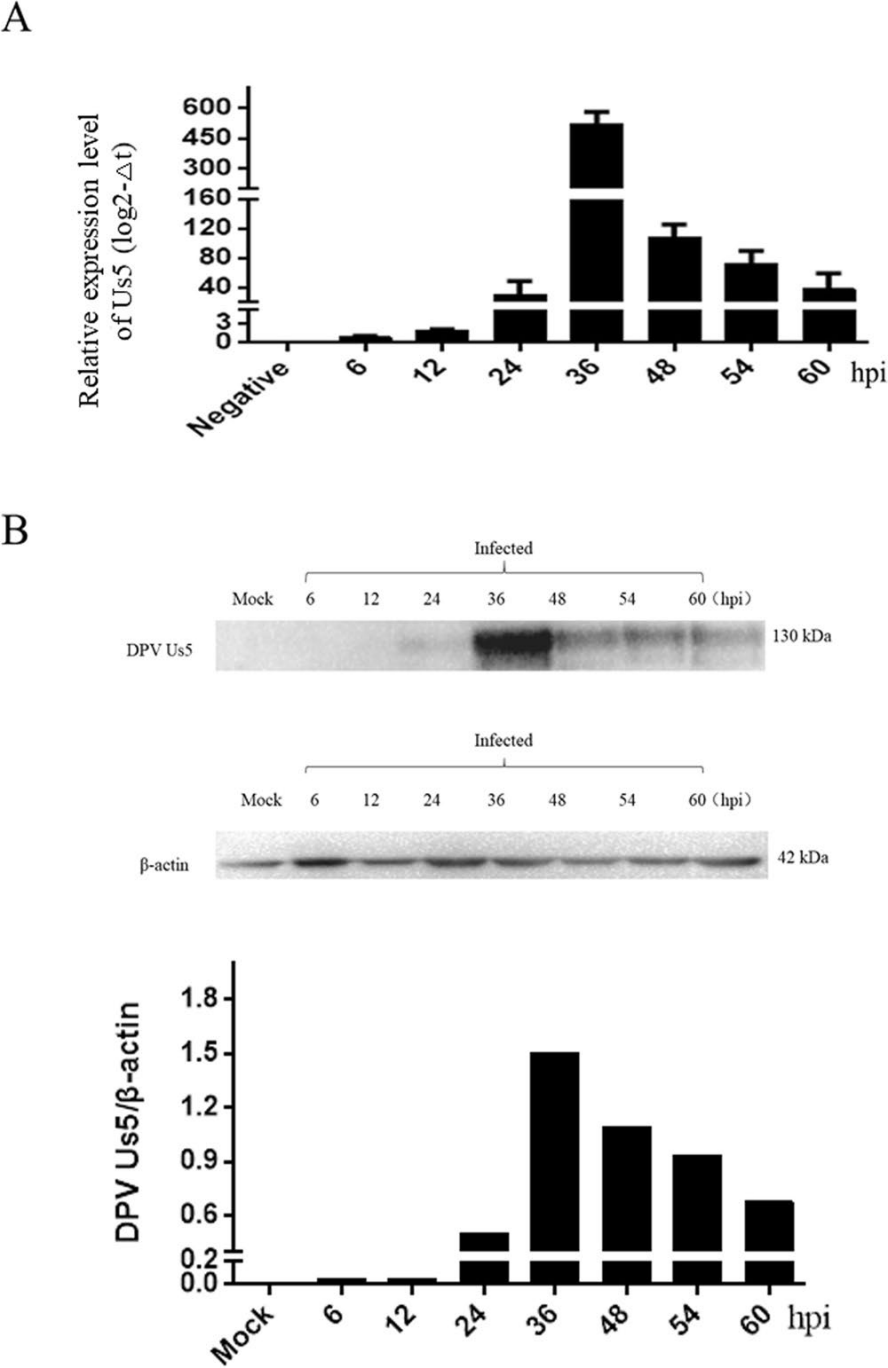
The transcription and expression kinetics of the DPV Us5 gene. (**A**) Relative transcript levels of the DPV Us5 gene at 6, 12, 24, 36, 48, 54, and 60 hpi. (**B**) Western blotting analysis for Us5 and β-actin at 6, 12, 24, 36, 48, 54, and 60 hpi (approximately 130 kDa) with anti-Us5 serum and anti-β-actin serum.

**Figure 2:**
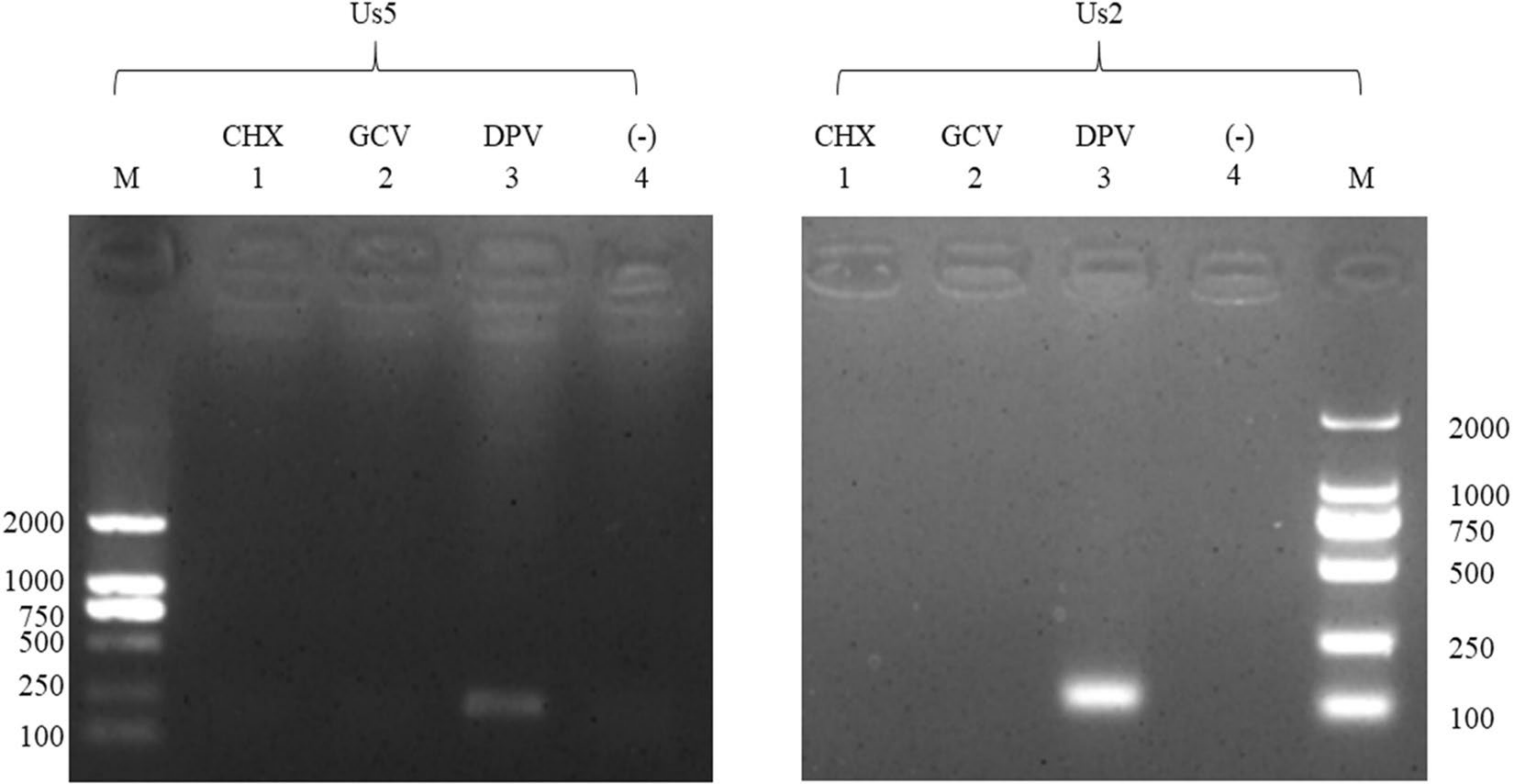
Pharmaceutical identification of virion gene type. M: DL2000 marker; 1: DEV-infected cells treated with 50 μg/ml cycloheximide (CHX); 2: DEV-infected cells treated with 300 μg/mL ganciclovir (GCV); 3: DPV-infected cells without drugs; 4: untreated DEF cells. The DPV Us5 gene (156 bp) and the L gene Us2 (111 bp) are shown.

The melting curves showed that the specificities of the primers were excellent, and standard curves were established to evaluate the efficiency of the assays (data not shown). The results showed that the Us5 gene transcript was detected at 6 hours post infection (hpi); Us5 expression gradually increased, peaking at 36 h, and then steadily decreased at 48 hpi, 54 hpi, and 60 hpi (Fig. 1A). As shown in Fig. 1B, a specific protein band of approximately 130 kDa was initially detected at 24 h. Peak expression occurred at 36 hpi, decreasing steadily thereafter, and Us5 was still detected at 60 hpi. The gene and protein expression levels of DPV Us5 are consistent with those of a late gene. To further verify the Us5 gene type, the protein synthesis inhibitor cyclohexamide (CHX) and the DNA polymerase inhibitor ganciclovir (GCV) were used to determine the kinetic class. Us5 (amplified length of 156 bp) and Us2 (amplified length of 111 bp, DPV late gene) were detected only when no drugs were present and there was no DPV infection (Fig. 2). Thus, Us5 is a late gene due to its strict dependence on the early synthesis of viral DNA and protein.

### Subcellular localization of DPV Us5

**Figure 3:**
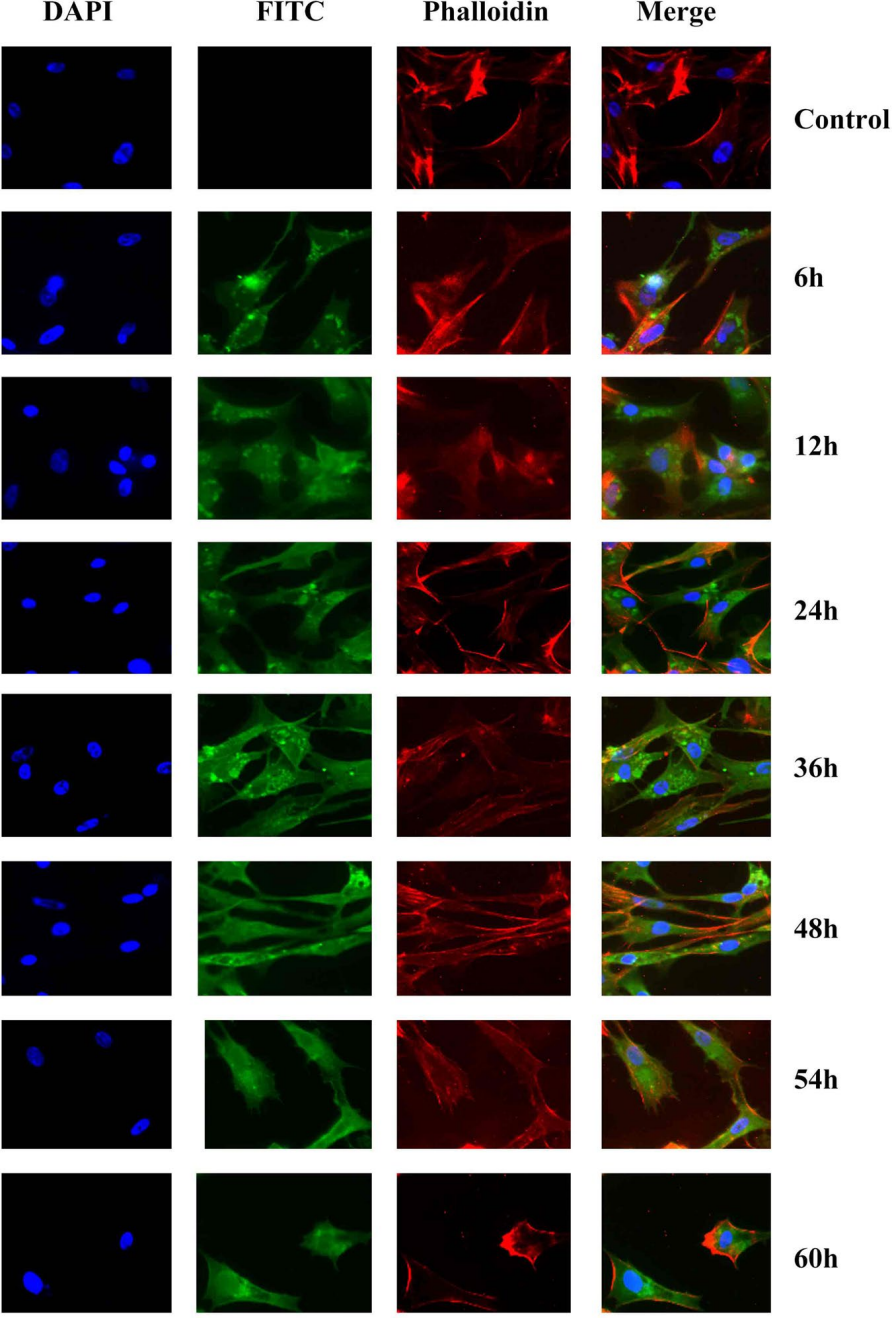
Dynamic intracellular localization of the Us5 protein during DPV infection at different times. Fluorescence microscopic images of Us5 localization (green in all images) at 6, 12, 24, 36, 48, 54 and 60 hpi. Nuclei are indicated in blue (DAPI) and cytoskeleton are indicated red (Phalloidin).

The results showed that Us5 protein-specific fluorescence, which is indicated in green (FITC), was localized primarily in the cytoplasm at 12 hpi. As time progressed, Us5 protein-specific fluorescence was localized mainly in the cytoplasm at 12 hpi, 24 hpi, 36 hpi, 48 hpi, 54 hpi, and 60 hpi. However, little Us5 protein-specific fluorescence was located in the perinuclear region at 36 hpi and 48 hpi. In contrast, no fluorescence was observed in the control groups. The nuclei are indicated in blue (DAPI), and cytoskeleton are indicated red (Phalloidin) (Fig. 3).

### The DPV Us5 protein is a component of the virion

**Figure 4:**
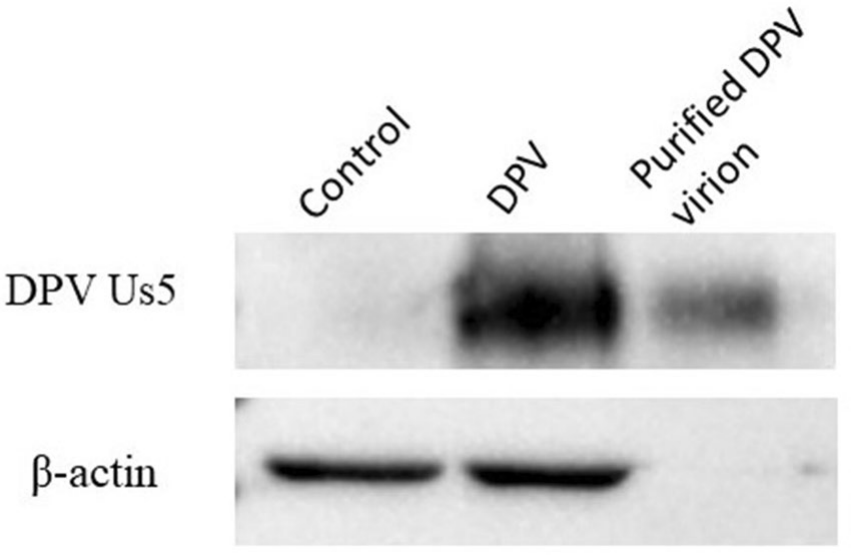
The Us5 protein is a component of the virion, as determined by Western blotting.

We identified 40 structural proteins in the purified DPV virions (data not shown), including the DPV Us5 protein. Two unique DPV Us5 peptides were detected, while three unique peptides matched DPV gC (P < 0.05). The relative abundance of Us5 was low based on the exponentially modified protein abundance index (emPAI) (Table 1). As shown in Fig. 4, the Us5 protein could be detected in DPV-infected cells and purified virions.

**Table 1:** Viral content of DPV extracellular virions (partial).

Protein	Description	Score	Mass	Matches	Sequences	emPAI	NCBI Accession
UL44	glycoprotein C	97	47,836	6 (3)	6 (3)	0.22	AJG04885.1
Us5	glycoprotein J	31	61,475	6 (2)	5 (2)	0.11	ADU04075.1

### H_2_O_2_ induces apoptosis of DEF cells

**Figure 5:**
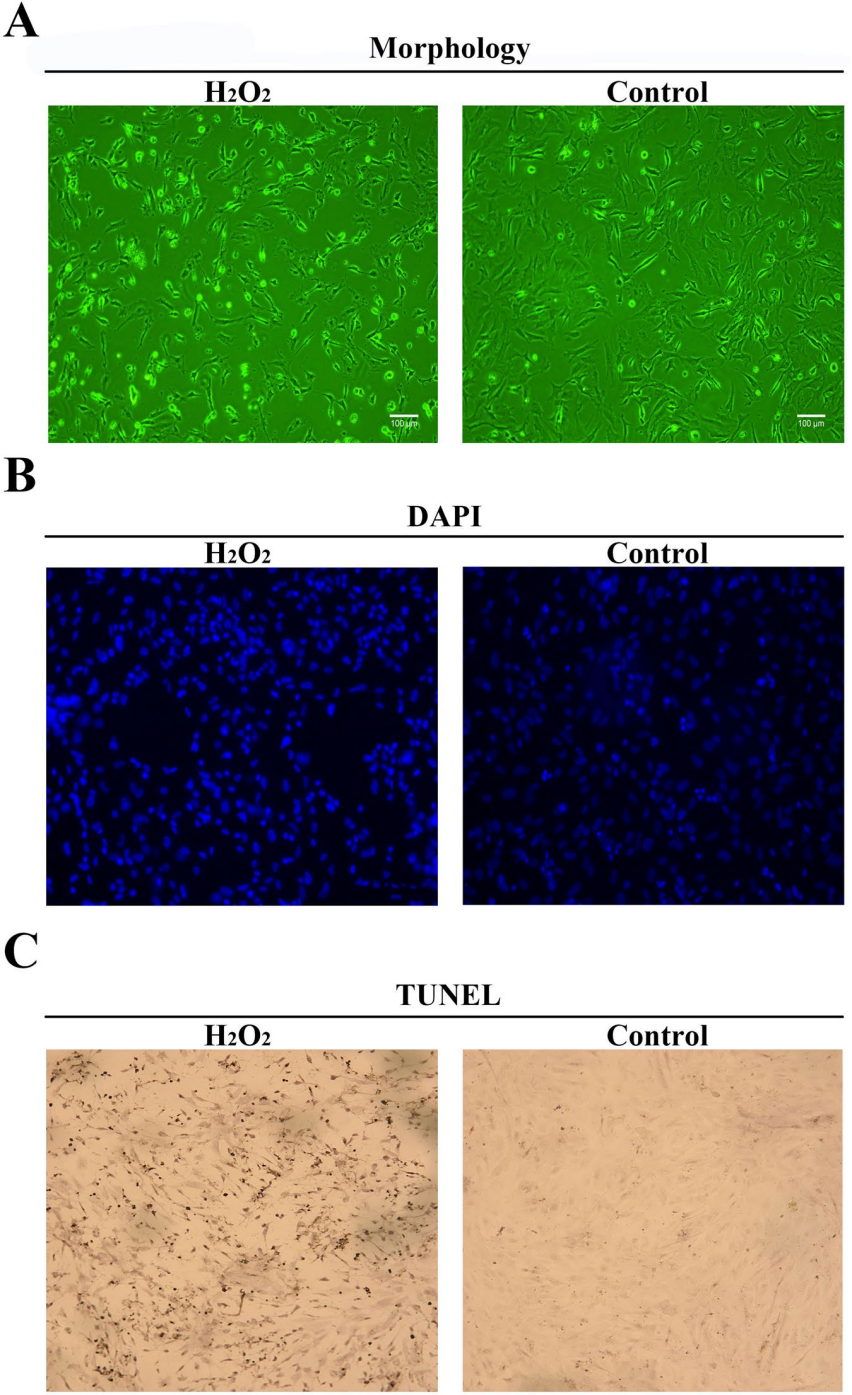
H_2_O_2_ induces apoptosis in DEF cells. (**A**) Cells were observed using phase contrast microscopy (×100). Non-H_2_O_2_-treated cells were used as controls (**B**). The cells were fixed and stained with DAPI. The arrows indicate apoptotic nuclei, and non-H_2_O_2_-treated cells were used as controls. (**C**) TUNEL assay. The arrows refer to TUNEL- positive cells, and non-H_2_O_2_-treated cells were used as controls.

**Figure 6:**
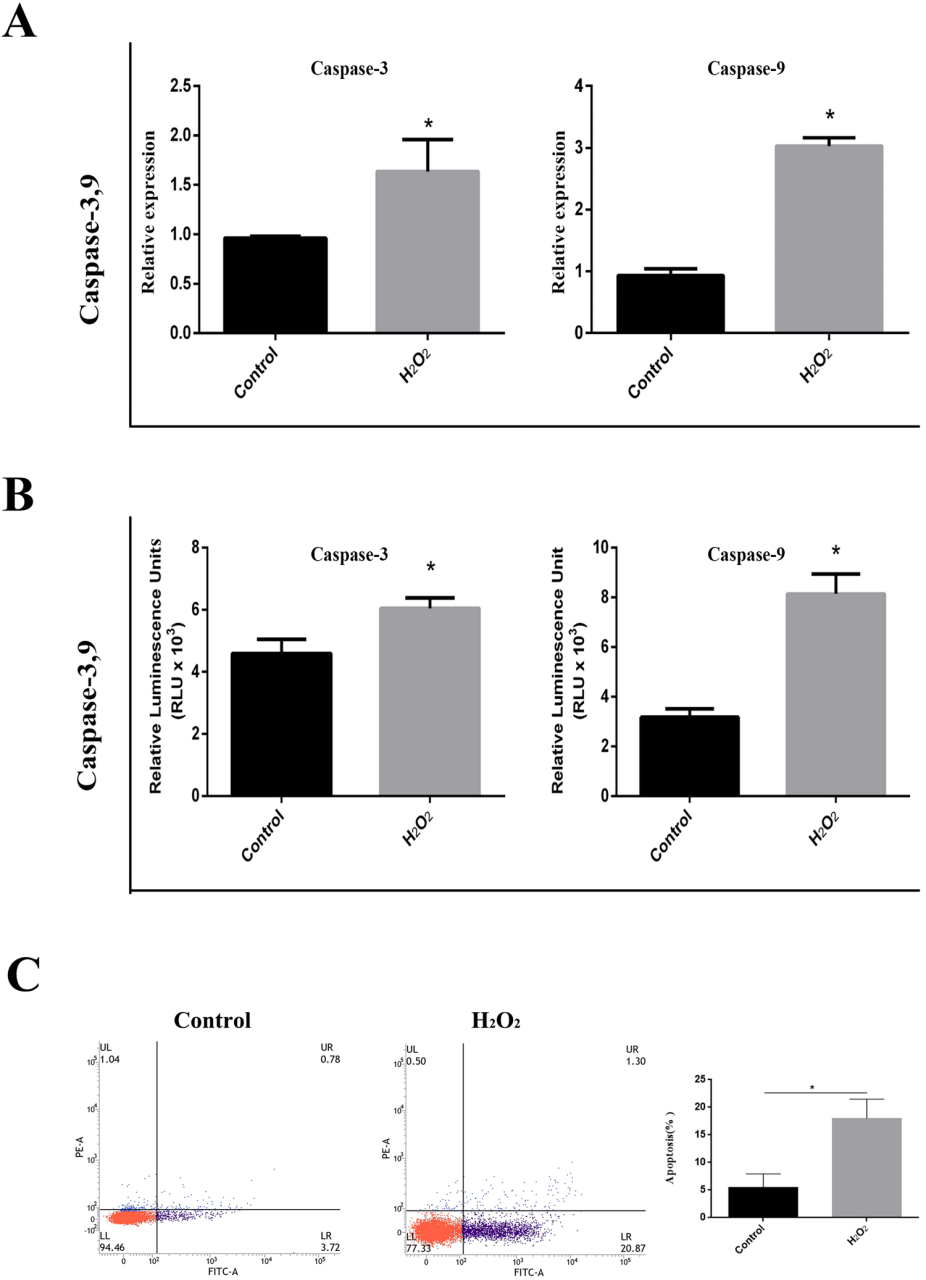
Activity of caspase-3 and caspase-9 induced by H_2_O_2_ in DEF cells. (**A**) The mRNA expression levels of caspase-3 and caspase-9 in H_2_O_2_-treated and non-H_2_O_2_-treated cells. (**B**) The activity of caspase-3/7 and caspase-9 in H_2_O_2_**-**treated and non-H_2_O_2_-treated cells. All data are presented as the means ± SD from three individual experiments. Non-H_2_O_2_-treated cells were used as controls. p* < 0.05 compared to the non-H_2_O_2_-treated controls. (**C**) Flow Cytometry results indicated that H_2_O_2_-treated cells have 22% apoptosis cells.

The results showed that the H_2_O_2_-treated cells were shrunken, detached, rounded and had lost adherence (Fig. 5A). Compared to those of untreated cells, the nuclei of H_2_O_2_-treated cells were smaller and distorted; in addition, chromatin was collected on the nuclear membrane and apoptotic bodies appeared (Fig. 5B). Positive TUNEL staining, a hallmark of apoptotic cell death, was detected in H_2_O_2_-treated cells, whereas non-H_2_O_2_-treated cells were negative for TUNEL staining (Fig. 5C). In addition, Flow Cytometry results indicated that H_2_O_2_-treated cells have 22% apoptosis cells (Fig. 6C).

### H_2_O_2_ activates caspase-3 and caspase-9 in DEF cells

The mRNA level of caspase-3 in H_2_O_2_-treated cells was approximately 1.65 times that in untreated cells, and the mRNA level of caspase-9 in H_2_O_2_-treated cells was approximately 3 times that in untreated cells (Fig. 6A). H_2_O_2_ treatment of cells led to a significant increase in the activity of the caspase-3/7 and caspase-9 proteins (Fig. 6B). The luminescence values of caspase-3/7 and caspase-9 in untreated cells were 4.5 × 10^3^ RLU and 3.0 × 10^3^ RLU per ten thousand cells, respectively. The luminescence value of caspase 3/7 and caspase 9 in treated cells were 6.0 × 10^3^ RLU and 8.0 × 10^3^ RLU per ten thousand cells, respectively (Fig. 6B).

### Expression of the recombinant plasmid pCAGGS-Us5 in DEF cells

**Figure 7:**
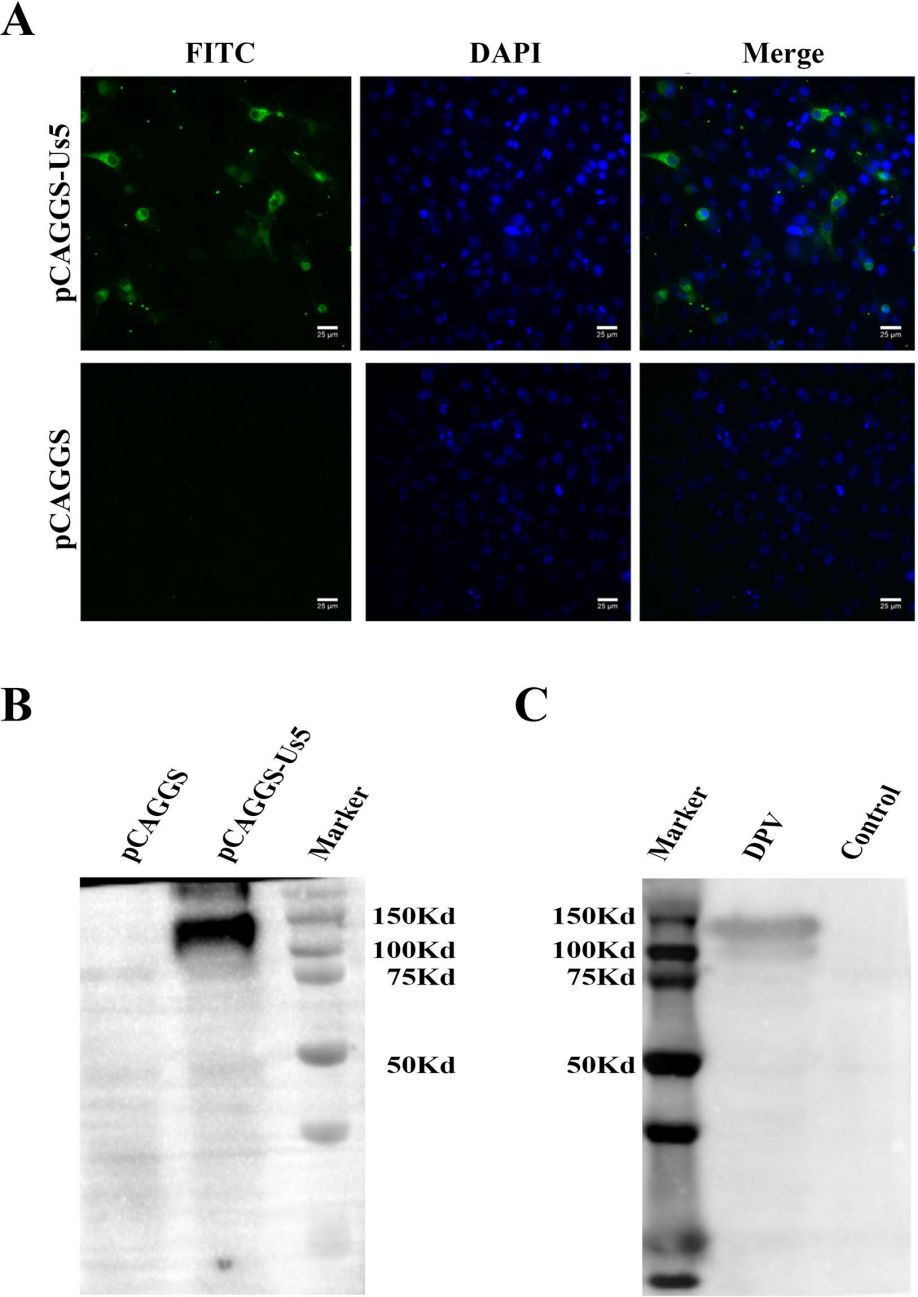
Expression of the Us5 protein in DEF cells. (**A**) Indirect immunofluorescence of DPV Us5 in DEF cells. Costaining was performed with a rabbit anti-DPV-Us5 polyclonal antibody and a fluorescein-conjugated goat anti-rabbit IgG antibody. (**B**) Western blotting of DPV Us5 in DEF cells. A rabbit anti-DPV-Us5 polyclonal antibody was used as the primary antibody. A goat anti-rabbit IgG (H + L)-HRP antibody was used as the secondary antibody. Noninfected cells were used as controls. (**C**) Western blotting of pCAGGS-Us5 in DEF cells. A duck anti-DPV polyclonal antibody was used as the primary antibody. A goat anti-duck IgG (H + L)-HRP antibody was used as the secondary antibody. Cells transfected with the pCAGGS plasmid were used as the controls.

pCAGGS and pCAGGS-Us5 plasmids were transfected into DEFs. In Fig. 7A, fluorescence is indicated in green (FITC) in pCAGGS-Us5 plasmid-transfected cells. The expression of the pCAGGS-Us5 plasmids was detected by Western blotting, which showed approximately 130 kDa bands (Fig. 7B). Similarly, the expression of DPV Us5 was detected, and the Western blot showed approximately 130 kDa bands (Fig. 7C).

### DPV Us5 inhibits apoptosis induced by H_2_O_2_ in DEF cells

**Figure 8:**
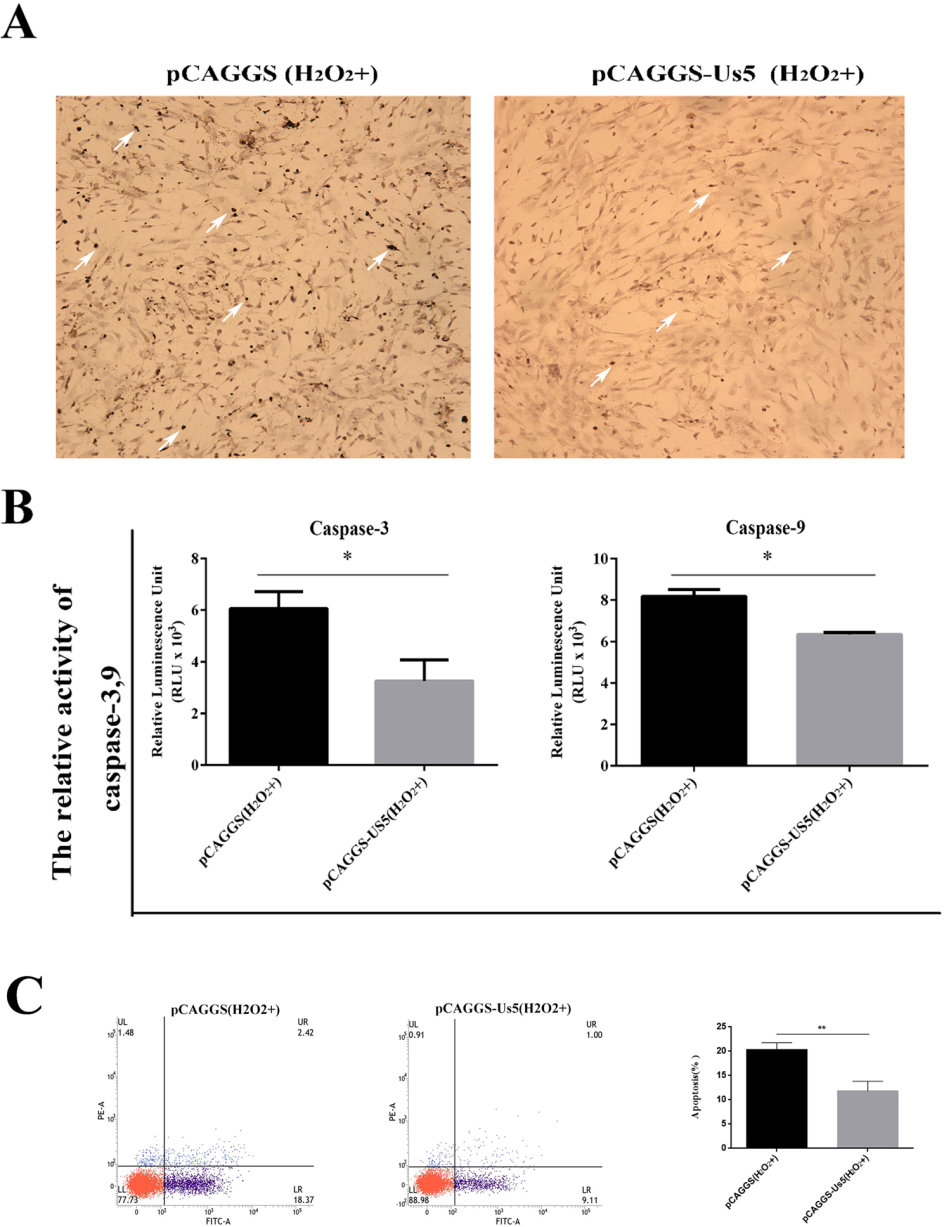
Effect of the DPV Us5 protein on H_2_O_2_-induced apoptosis. (**A**) TUNEL assay. The arrows indicate TUNEL-positive cells. Cells transfected with the pCAGGS plasmid and treated with H_2_O_2_ were used as controls. (**B**) The activity of caspase-3/7 and caspase-9 in cells transfected with the pCAGGS-Us5 plasmid and treated with H_2_O_2_ and cells transfected with the pCAGGS plasmid and treated with H_2_O_2_. (**C**) H_2_O_2_-treated cells transfected with the pCAGGS-Us5 plasmid cloud inhibit a lot of apoptosis. All data are presented as the means ± SD from three individual experiments.  p* < 0.05, p** < 0.01 compared to the non-Us5-expressing control.

Compared with that of H_2_O_2_-treated cells transfected with the pCAGGS plasmid, the positive TUNEL staining of H_2_O_2_-treated cells transfected with the pCAGGS-Us5 plasmid was significantly reduced (Fig. 8A). The results showed that the caspase-3/7 protein activity in H_2_O_2_-treated cells transfected with the pCAGGS plasmid was approximately 6 × 10^3^ RLU per ten thousand cells, while that of H_2_O_2_-treated cells transfected with the recombinant plasmid pCAGGS-Us5 was approximately 3.3 × 10^3^ RLU per ten thousand cells (Fig. 8B). The results also showed that the caspase-9 protein activity of H_2_O_2_-treated cells transfected with the pCAGGS plasmid was approximately 8.1 × 10^3^ RLU per ten thousand cells, and that of H_2_O_2_-treated cells transfected with the recombinant plasmid pCAGGS-Us5 was approximately 6 × 10^3^ RLU per ten thousand cells (Fig. 8B). As is shown in Fig. 8C, compared with that of H_2_O_2_-treated cells transfected with the pCAGGS plasmid, H_2_O_2_-treated cells transfected with the pCAGGS-Us5 plasmid cloud inhibit a lot of apoptosis.

### Anti-apoptosis ability of DPV CHv-BAC parental viruses, DPV CHv-BAC-ΔgJ mutant viruses and CHv-BAC-ΔgJR rescue viruses

**Figure 9:**
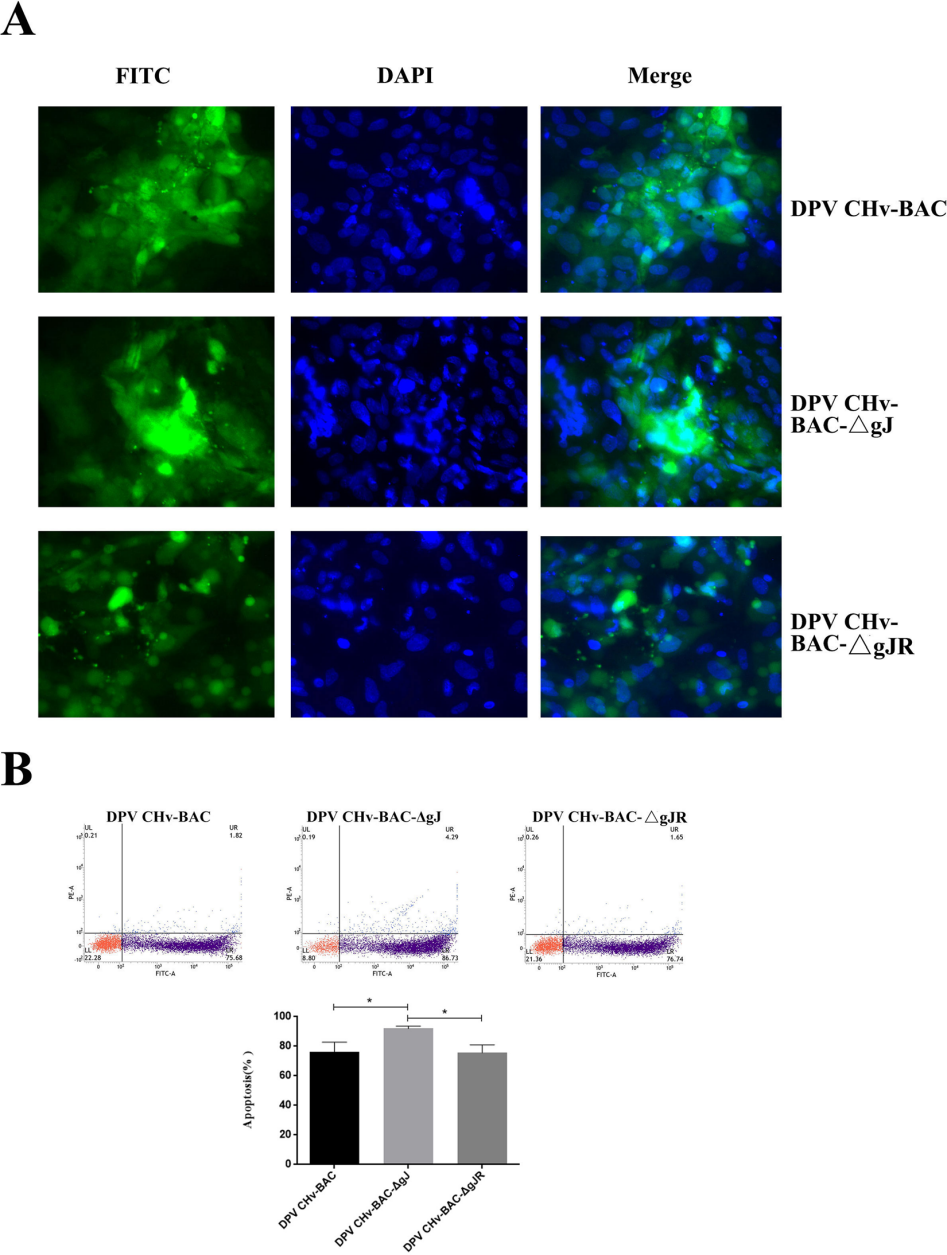
Anti-apoptosis ability of DPV CHv-BAC parental viruses, DPV CHv-BAC-ΔgJ mutant viruses and CHv-BAC-ΔgJR rescueviruses. (**A**) The apoptosis DEF cells of recombination viruses relative to the parental virus were investigated at 48 hpi utilizing DAPI staining. (**B**) The apoptosis DEF cells of recombination viruses relative to the parental viruses were investigated at 48 hpi utilizing Flow Cytometry. All data are presented as the means ± SD from three individual experiments. p* < 0.05 compared to the DPV CHv-BAC parental viruses and the DPV CHv-BAC-ΔgJR rescue viruses.

According to the following results, we further explored whether Us5 could inhibit apoptosis induced in DPV infected cells. There have been reported that DPV CHv can cause DEF cells apoptosis^24^, and we constructed the DPV CHv-BAC-ΔgJ mutant viruses successfully^23^. Therefore, the DEF cells were infected with DPV-CHv, DPV CHv-BAC-ΔgJ mutant viruses and DPV CHv-BAC-ΔgJR rescue viruses respectively. The apoptosis DEF cells of recombination viruses relative to the parental virus were investigated at 48 hpi utilizing DAPI staining. As is shown in the Fig. 9A, the DPV CHv-BAC parental virus and the DPV CHv-BAC-ΔgJR rescue viruses induced apoptosis DEF cells that were similar in number. The apoptosis cells induced by DPV CHv-BAC-ΔgJ mutant viruses more than the parental virus. To better assess the apoptosis cells induced by different viruses, Flow Cytometry was analyzed the DEF Cells of apoptosis, this analysis also confirmed that the apoptosis cells induced by DPV CHv-BAC-ΔgJ mutant viruses more than the parental viruses (Fig. 9B).

## Discussion

Until now, reports about the DPV Us5 gene have been limited. Our group constructed two different mutated viruses, a gJ deleted mutant, ΔgJ, and a rescue virus with gJ restored, ΔgJR, showing that the gJ was slightly impaired in viral replication, virion assembly and cell-to-cell spread and was not essential in virion envelopment^23^. In this study, we further studied the function of DPV pUs5 as the first step in exploring the molecular characterization and antiapoptotic function of the DPV Us5 protein.

During herpesvirus infection, viral genes are expressed sequentially in three distinctly defined stages: immediate early gene (IE), early gene (E), and late gene (L). The IE genes are immediately transcribed upon infection, without interaction with other proteins^25^. The E genes are transcribed prior to viral DNA replication in an IE protein-dependent manner. Transcription of the L genes begins after the synthesis of DNA, and viral protein translation begins^7,26^. HSV-1 Us5 is a late gene, with partial dependency on DNA replication for its expression^21^. The current study demonstrates that Us5 is similar to other HSV glycoproteins in that it is regulated as a late gene. To identify the DPV Us5 gene type, we used Western blot and RT-PCR analyses to study the expression and transcriptional kinetics of DPV Us5 at different time points after infection. Based on our group of previous studies^27–31^, we speculated that accumulation of the DPV Us5 protein may occur during the late stage of infection. The IE genes are expressed in the presence of the protein synthesis inhibitor CHX, while the transcription of the E and L genes is inhibited in the presence of CHX. To further study DPV Us5 gene, we treated infected cells with a protein synthesis inhibitor, CHX, and a DNA polymerase inhibitor, ganciclovir (GCV), and observed that the Us5 gene was inhibited in both the CHX and GCV groups.

Intracellular viral protein localization is associated with viral protein function, but intracellular DPV Us5 localization has thus far remained unclear. A previous publication demonstrated that Us5 was located on the surface of HSV-1-infected cells. Further studies showed that Us5, similar to other HSV glycoproteins, was mainly found in intracellular membranes, especially in Golgi bodies, the endoplasmic reticulum and endosomes^21^. In this study, DPV infection in DEFs was detected by IFA. The results showed that DPV Us5 was mainly localized in the cytoplasm. Then, the recombinant plasmid pCAGGS-Us5 was transfected into DEF cells and revealed that Us5 was localized mainly in the cytoplasm. This finding indicates that Us5 modulates cellular functions occurring in the cytoplasm. DPV Us5 is a glycoprotein that may be differentially glycosylated to form different glycoproteins during posttranslational modification. The recombinant plasmid pCAGGS-Us5 was transfected into DEF cells and detected by Western blotting, which showed one specific band of approximately 130 kDa. Interestingly, the expression of Us5 after DPV infection in DEFs, as detected by Western blotting, also showed one specific band of approximately 130 kDa. The size of the Us5/gp2 protein encoded by the α-herpesvirus Us5 varies greatly among different viruses. The gp2 gene of equine herpesvirus-1 (EHV-1) is homologous to the Us5 gene and expresses three proteins with different molecular weights, 230 kDa, 65 kDa and 42 kDa^11^; the Us5 and gD proteins of HSV-1 and the gG protein of Macacine herpesvirus-1 also have different molecular weights^21,32^.

Viruses infect cells to facilitate their own breeding and to ensure their long-term survival in host cells, and viruses can evolve the necessary functions to prevent apoptosis and delay cell death. This ability to evolve is one of the biological characteristics of α-herpesviruses^33^. Many studies have confirmed that α-herpesviruses encode proteins with antiapoptotic functions^16–18,34–36^. During the early stage of infection, these genes promote the survival of host cells, facilitate the replication of the virus itself and facilitate the production of subviruses. One of the functions reported for the Us5 orthologs of HSV-1 is the inhibition of apoptosis induced by viral infection and exogenous factors^18,37^. To study the antiapoptotic functions of DPV Us5 gene encoding protein, we first established an apoptotic model in which the apoptosis of DEF cells was induced by H_2_O_2_. There are many ways to induce cell apoptosis *in vitro*, such as with UV, sorbitol, and H_2_O_2_, among other methods^38–41^, and H_2_O_2_ has been used to establish apoptosis models in multiple cell types for antiapoptotic function experiments^42–44^. The morphology of the cells changed after treatment with 400 μM H_2_O_2_. These changes are consistent with the characteristics of apoptosis^45^. In addition to examining morphology, detecting apoptotic factors is another classic way to verify apoptosis. In the apoptotic cascade, caspases, including caspase-3, are the major effectors of apoptosis^46^. Therefore, to further verify that H_2_O_2_ induces DEF apoptosis, detection of the mRNA levels and activity of the apoptotic factor caspase-3 was necessary. In the current experiment, all of the data showed that the caspase-3 mRNA levels and activity were increased in H_2_O_2_-treated cells relative to those in control cells. These results prove the feasibility of hydrogen peroxide-induced DEF cells apoptosis. H_2_O_2_ induces apoptosis in various ways; including by increasing intracellular ROS levels. Excess intracellular ROS cause mitochondrial dysfunction, triggering the mitochondrial apoptotic pathway. This pathway activates cytochrome C, Apaf-1 and other factors and leads to the cleavage of downstream caspase-9, which activates other caspases, resulting in apoptosis^22,47–49^. In this study, the mRNA and activity levels of caspase-9 in H_2_O_2_-treated cells were significantly increased compared to those in control cells, suggesting that H_2_O_2_-induced apoptosis in DEF cells may be related to the mitochondrial pathway. This study is the first to show that DPV Us5 is necessary for the effective protection of cells against apoptosis triggered by H_2_O_2_. The results showed that the expression of the Us5 gene encoding protein inhibits apoptosis induced by H_2_O_2_ and inhibits the H_2_O_2_-induced activation of caspase-3/7 and caspase-9, suggesting that the Us5 gene may inhibit the intrinsic pathway. The DPV CHv-BAC parental viruses and the DPV CHv-BAC-ΔgJR rescue viruses induces apoptosis DEF cells that were similar in number. The apoptosis cells induced by DPV CHv-BAC-ΔgJ mutant viruses more than the parental viruses. However, some studies have shown that Us5 can inhibit the extrinsic pathway. In one study, the removal of Us5 from HSV-1 significantly abolished protection against Fas-mediated apoptosis and partially reduced protection against UV radiation by inhibiting caspase-3/8 activation^50^. In addition transfecting Us5 has been found to inhibit Fas/UV-induced apoptosis and to weaken the activation of caspases^18^. Overall, the results suggested that Us5 is sufficient to establish an antiapoptotic phenotype. Us5-deleted HSV-1 shows a weakened protective effect against cytotoxic T lymphocyte-induced apoptosis, and gJ is sufficient to inhibit F_0_F_1_ ATP synthase activity and the production of ROS to inhibit apoptosis^51^. However, the mechanism by which DPV Us5 blocks apoptosis remains elusive. Others should further study the underlying mechanism through the use of additional apoptosis models other than H_2_O_2_-induced apoptosis.

In summary, this study found that the DPV Us5 gene is a late gene and that the Us5 protein is a component of the virion, is localized mainly in the cytoplasm, and can inhibit apoptosis induced by H_2_O_2_.

## Materials and Methods

### Virus, cells, and other significant materials

The CHv strain of DPV (GenBank accession number: JQ647509), DPV CHv-BAC parental viruses, DPV CHv-BAC-ΔgJ mutant viruses, CHv-BAC-gJR mutant viruses *E*. *coli* DH5α cells, the prokaryotic expression vector pCAGGS, the recombinant β-actin plasmid, duck anti-DPV serum, and rabbit anti-Us5 serum were preserved and provided by our laboratory. The DEF monolayer was incubated at 37 °C with 5% CO_2_ in Minimal Essential Medium (MEM, Gibco) containing 10% newborn calf serum (NBS, Gibco) and 100 μg/mL streptomycin. After DPV infection, the cells were cultured in MEM supplemented with 3% NBS. DPV operation under BSL-2.

### Plasmid construction and transfection

The Us5 gene fragment was ligated into the pCAGGS plasmid by homologous recombination using a ClonExpress® II One Step Cloning Kit. DEFs were transiently transfected in 6-well plates with 2.5 μg of the pCAGGS-Us5 recombinant plasmid using 5 μl of Lipofectamine 3000 transfection reagent (Thermo Scientific, Massachusetts, USA) according to the manufacturer’s guidelines. At 48 h after transfection, the supernatants of the DEF lysates were collected and stored at −80 °C until further use. The control group included the supernatant collected from DEFs transfected with the empty pCAGGS plasmid. The expression levels of the pCAGGS-Us5 plasmids were confirmed by Western blotting. The primers were designed with software of PRIMER 5 and are listed in Table 2.

**Table 2:** Primers.

Primer	Sequence (5′ → 3′)	Product (bp)
Us5 amplification primers F	CATCATTTTGGCAAAGAATTCGCCACCATGGCCATGTATACAGACGTTACGGTC	1701
Us5 amplification primers R	TTGGCAGAGGGAAAAAGATCTTCAAGCGTAATCTGGAACATCGTATGGGTATACCATACAAAGGCATA	
Us5 identified primers F	AATGTCATGTCGCAACGCTAGATA	156
Us5 identified primers R	TGTCCGCCACAGTCTGATTGATA	
Caspase-3 qPCR primers F	TGGTGTTGAGGCAGACAGTGGA	113
Caspase-3 qPCR primers R	CATTCCGCCAGGAGTAATAGCC	
Caspase-9 qPCR primers F	GCTGCTTCAACTTCCTCCGTA	166
Caspase-9 qPCR primers R	CATCTCCACGGACAGACAAAGG	
β-actin qPCR primers F	CGGGCATCGCTGACA	177
β-actin qPCR primers R	GGATTCATCATACTCCTGCTTTGCT	
Us2 primers F	AGACGGTTCCGAAAGTACAG	111
Us2 primers R	TCGGCAGCACCAATAATCC	

### Isolation of RNA and analysis of Us5, caspase-3, and caspase-9 mRNA expression by RT-PCR

Total RNA was extracted from DPV-infected DEFs at different time points post infection (6 h, 12 h, 24 h, 36 h, 48 h, 54 h, and 60 h). The DEFs included H_2_O_2_-treated cells and mock-treated cells as well as transfected and nontransfected H_2_O_2_-treated cells, and the total RNA was extracted using RNAiso Plus (TaKaRa, Dalian, China) according to the manufacturer’s instructions. Extracted RNA was immediately reverse transcribed into first-strand cDNA using a PrimeScript RT Reagent Kit with gDNA Eraser (TaKaRa, Dalian, China). For RT-PCR, a 20 µL total reaction volume was used that contained 10 µL of SYBR Premix Ex Taq II (Tli RNaseH Plus), 1 µL of forward primer, 1 µL of reverse primer, 6 µL of RNase-free water and 2 µL of cDNA. The thermal cycling program included an initial denaturation step for 30 seconds at 95 °C followed by 40 cycles of 5 seconds at 95 °C and 30 seconds at the melting temperature of the specific primer pair. Triplicate experiments were performed to analyze the gene expression of Us5 and β-actin, and the relative transcription level of the DPV Us5 gene was calculated using the 2^−ΔCt^ method simplified from the 2^−ΔΔCt^ method^28^. The relative expression levels of caspase-3 and caspase-9 were determined using β-actin as an endogenous control with the comparative Ct (−2^ΔΔin^) method and a real-time thermal cycler (CFX96 Bio-Rad, Hercules, CA, USA)^52^. The primers were designed with PRIMER 5 software and are listed in Table 2.

### Pharmacological inhibition tests

Pharmacological inhibition tests were performed to confirm the kinetics of DPV Us5^27,53^. Four bottles of DEFs were prepared. Three were inoculated with DPV; one bottle contained no drugs, one bottle contained 300 μg/mL GCV (a DNA polymerase synthesis inhibitor), and one bottle contained 100 μg/mL CHX (a protein synthesis inhibitor). The fourth bottle contained untreated mock-infected cells. The infected cells were harvested 24 h after infection and washed twice with PBS. Total RNA was extracted and cDNA was synthesized as described above for RT-PCR. The Us5 gene type was identified by PCR (Us2 was used as a late gene control)^29^. The primers were designed with PRIMER 5 software and are listed in Table 2.

### Us5 expression analysis by Western blotting

Western blotting was conducted as described in a previous study^54^. DEFs transfected with the empty pCAGGS plasmid and the pCAGGS-Us5 plasmid were collected at 48 h, and the protein expression was confirmed by Western blotting. A duck anti-DPV polyclonal antibody (at a final dilution of 1:500) was used as the primary antibody, and a goat anti-duck IgG antibody (at a final dilution of 1:2000) was used as the secondary antibody. In addition, DPV-infected cells and noninfected cells were collected at 6 h, 12 h, 24 h, 36 h, 48 h, 54 h, and 60 h, and the protein expression was confirmed by Western blotting^27^. A rabbit anti-DPV-Us5 polyclonal antibody (at a final dilution of 1:1000) was used as the primary antibody, and a goat anti-rabbit IgG antibody (at a final dilution of 1:3000) was used as the secondary antibody.

### Subcellular localization of DPV Us5 by IFA

IFA was conducted using a standard procedure^27^. Briefly, pCAGGS-Us5 plasmid-transfected cells and pCAGGS plasmid-transfected cells were collected at 48 h, and DPV-infected cells and noninfected cells were collected at 6 h, 12 h, 24 h, 36 h, 48 h, 54 h, and 60 h. The cells were fixed with 4% paraformaldehyde in PBS for 30 min, washed three times with PBST, permeabilized with 0.25% Triton X-100 in PBS for 30 min at 4 °C and blocked for 1 h with 5% BSA PBS at 37 °C. Additionally, the cells were incubated for 1 h with the rabbit anti-DPV-Us5 polyclonal antibody at a 1:400 dilution at 37 °C. After the cells were washed, a 1:1000 dilution of goat anti-rabbit IgG-FITC secondary antibody was prepared in 1% BSA PBS and incubated with the cells for 1 h at 37 °C. Then, the cell nuclei were stained with DAPI for 15 min at room temperature. Finally, The cytoskeleton were stained with Phalloidin for 30 min at room temperature. After the coverslips were sealed on glass slides with glycerin buffer, the cells were examined with a confocal microscope (Nikon A1, Japan).

### DAPI staining of nuclei

DAPI staining of nuclei was conducted using a standard procedure^27^. Briefly, H_2_O_2_-treated cells and non-H_2_O_2_-treated cells were collected at 12 h and fixed with 4% paraformaldehyde for 60 min. The solution was permeabilized for 30 min and stained with DAPI for 10 min. The nuclei were examined with a confocal microscope (Nikon A1, Japan).

### Apoptosis of DEFs detected by TUNEL assay

Apoptosis was detected using an *in situ* cell apoptosis detection kit (POD) (Boster Biological Technology, USA). Briefly, cells were fixed with 4% paraformaldehyde for 60 min and incubated with the terminal deoxynucleotidyl transferase-mediated dUTP-biotin nick end labeling (TUNEL) staining reaction mixture containing the enzyme solution and label solution prepared according to the manufacturer’s protocol. The chromogenic substrate 3,3′-diaminobenzidine (DAB) was then added to visualize the TUNEL staining. The apoptotic nuclei were yellow, and the apoptotic cells were observed under the microscope.

### Caspase-3, caspase-7, and caspase-9 activity detection with a Caspase-Glo 3/7 assay kit and a Caspase-Glo 9 assay kit

The activity of caspase-3/7 and caspase-9 was measured using a Caspase-Glo 3/7 assay kit and a Caspase-Glo 9 assay kit, respectively (Promega, Madison, Wisconsin, USA), according to the manufacturer’s instructions. Briefly, after the treated adherent cells were removed, the cells were collected into EP tubes. The cells were counted with a blood cell counting plate. From each sample, approximately 10,000 cells were added to 100 µL of Caspase-Glo reagent in a 96-well white-wall plate and incubated for 30 min. Luciferase activity was detected with a multifunctional microplate reader (Thermo Scientific, Massachusetts, USA).

### Virion purification

DPV-infected DEFs were collected at 48 h and purified by low-speed centrifugation (2000 × g, 20 min, 4 °C) to remove the cell debris. Extracellular DPV virions were harvested by ultracentrifugation (40,000 × g, 2 h, 4 °C) through a 30% (wt/vol) sucrose cushion in a Beckman Ti70 rotor. The band containing virions was collected by isopycnic gradient ultracentrifugation in a continuous 30 to 60% (wt/vol) potassium tartrate gradient in TBS (40,000 × g, 2 h, 4 °C) in a Beckman SW60 rotor, diluted ten-fold in TBS and then pelleted by ultracentrifugation (20,000 × g, 60 min, 4 °C). Finally, the pellet was resuspended in TBS and stored at −80 °C.

### Mass spectrometry

Purified virion samples were separated by 12% SDS-PAGE. The gel was stained with Coomassie brilliant blue (Sigma-Aldrich) and sent to Sangon Biotech Company (Shanghai, China) for liquid chromatography-tandem mass spectrometry (LC-MS/MS) analysis^27^. The details of LC-MS/MS, in-gel trypsin digestion, and the database searches were the same as those described in a previous study^55–57^.

### Flow cytometric analysis of apoptosis

The apoptotic cells were detected by flow cytometry (FCM); 5 µL of Annexin V-Fluorescein isothiocyanate (V-FITC) (BD Pharmingen, USA, 51–65874X) and 5 µL of propidium iodide (PI) (BD Pharmingen, USA, 51-66211E) were added to 100 µL of cell suspension and were incubated at 25 °C in the dark for 15 minutes. Annexin binding buffer (450 µL) (BD Pharmingen, USA, 51-66121E) was added to the mixture, and the percentage of apoptotic cells was assayed by FCM within 1 hour. Cell cycle was detected by FCM.

### Statistical analysis

The data are expressed as the means ± S.D. Statistical analysis was performed with Student’s t-test (GraphPad Prism 6); *p < 0.05 and **p < 0.01 indicate statistical significance compared with the control.

## References

[1] Xiang J (2012). Computational identification of microRNAs in Anatid herpesvirus 1 genome. Virol J.

[2] Wu Y (2012). Comparative genomic analysis of duck enteritis virus strains. J Virol.

[3] Wu Y (2012). Complete genomic sequence of Chinese virulent duck enteritis virus. J Virol.

[4] Shen CJ (2009). Identification and characterization of the duck enteritis virus UL51 gene. Arch Virol.

[5] Shen C (2009). Characterization of subcellular localization of duck enteritis virus UL51 protein. Virol J.

[6] Lu L (2010). Polyclonal antibody against the DPV UL46M protein can be a diagnostic candidate. Virol J.

[7] Lian B (2010). Identification and characterization of duck plague virus glycoprotein C gene and gene product. Virol J.

[8] Gou YF (2005). Morphological observation of virulent duck enteritis virus strain CH-Infected duck embryo fibroblasts by transmission electron microscopy. Chinese Journal of Veterinary Science.

[9] Jovasevic V, Roizman B (2010). The novel HSV-1 US5-1 RNA is transcribed off a domain encoding US5, US4, US3, US2 and alpha22. Virol J.

[10] Crabb BS, Allen GP, Studdert MJ (1991). Characterization of the major glycoproteins of equine herpesviruses 4 and 1 and asinine herpesvirus 3 using monoclonal antibodies. J Gen Virol.

[11] Mahmoud HY (2013). Characterization of glycoproteins in equine herpesvirus-1. J Vet Med Sci.

[12] Fuchs W, Wiesner D, Veits J, Teifke JP, Mettenleiter TC (2005). *In vitro* and *in vivo* relevance of infectious laryngotracheitis virus gJ proteins that are expressed from spliced and nonspliced mRNAs. J Virol.

[13] Bevilacqua F (1995). Construction of a herpes simplex virus/varicella-zoster virus (HSV/VZV) thymidine kinase recombinant with the pathogenic potential of HSV and a drug sensitivity profile resembling that of VZV. J Gen Virol.

[14] Eisenberg RJ (2012). Herpes virus fusion and entry: a story with many characters. Viruses.

[15] Mundt A, Mundt E, Hogan RJ, Garcia M (2011). Glycoprotein J of infectious laryngotracheitis virus is required for efficient egress of infectious virions from cells. The Journal of general virology.

[16] Wang X, Patenode C, Roizman B (2011). US3 protein kinase of HSV-1 cycles between the cytoplasm and nucleus and interacts with programmed cell death protein 4 (PDCD4) to block apoptosis. Proceedings of the National Academy of Sciences.

[17] Sciortino MT (2008). Involvement of gD/HVEM interaction in NF-kB-dependent inhibition of apoptosis by HSV-1 gD. Biochemical pharmacology.

[18] Jerome KR (2001). HSV and glycoprotein J inhibit caspase activation and apoptosis induced by granzyme B or Fas. J Immunol.

[19] Benetti L, Munger J, Roizman B (2003). The herpes simplex virus 1 US3 protein kinase blocks caspase-dependent double cleavage and activation of the proapoptotic protein BAD. J Virol.

[20] Benetti L, Roizman B (2007). In transduced cells, the US3 protein kinase of herpes simplex virus 1 precludes activation and induction of apoptosis by transfected procaspase 3. Journal of virology.

[21] Aubert M (2008). The antiapoptotic herpes simplex virus glycoprotein J localizes to multiple cellular organelles and induces reactive oxygen species formation. J Virol.

[22] Zhao C (2018). Programmed cell death: the battlefield between the host and alpha-herpesviruses and a potential avenue for cancer treatment. Oncotarget.

[23] You Y (2018). Duck plague virus Glycoprotein J is functional but slightly impaired in viral replication and cell-to-cell spread. Sci Rep.

[24] Guo Y (2009). Anatid herpesvirus 1 CH virulent strain induces syncytium and apoptosis in duck embryo fibroblast cultures. Vet Microbiol.

[25] Liu, C. *et al*. Duck enteritis virus UL54 is an IE protein primarily located in the nucleus. *Virology Journal* **12**, 198, 10.1186/s12985-015-0424-z (2015). 10.1186/s12985-015-0424-z PMC4658773 26606920

[26] Gruffat H, Marchione R, Manet E (2016). Herpesvirus Late Gene Expression: A Viral-Specific Pre-initiation Complex Is Key. Front Microbiol.

[27] He T (2018). Molecular characterization of duck enteritis virus UL41 protein. Virol J.

[28] Zhang D (2017). Molecular characterization of the duck enteritis virus US10 protein. Virol J.

[29] Gao J (2015). Identification and characterization of the duck enteritis virus (DEV) US2 gene. Genet Mol Res.

[30] Xie W (2010). Molecular cloning and characterization of the UL31 gene from duck enteritis virus. Mol Biol Rep.

[31] Zhang S (2011). Characterization of duck enteritis virus UL53 gene and glycoprotein K. Virol J.

[32] Perelygina L, Patrusheva I, Vasireddi M, Brock N, Hilliard J (2015). B Virus (Macacine herpesvirus 1) Glycoprotein D Is Functional but Dispensable for Virus Entry into Macaque and Human Skin Cells. Journal of virology.

[33] You, Y. *et al*. The suppression of apoptosis by α-herpesvirus. *Cell Death & Disease* **8**, e2749–e2749, 10.1038/cddis.2017.139 (2017). 10.1038/cddis.2017.139 PMC5477576 28406478

[34] Pontes MS, Van Waesberghe C, Nauwynck H, Verhasselt B, Favoreel HW (2016). Pseudorabies virus glycoprotein gE triggers ERK1/2 phosphorylation and degradation of the pro-apoptotic protein Bim in epithelial cells. Virus Res.

[35] Hagglund R, Munger J, Poon AP, Roizman B (2002). U(S)3 protein kinase of herpes simplex virus 1 blocks caspase 3 activation induced by the products of U(S)1.5 and U(L)13 genes and modulates expression of transduced U(S)1.5 open reading frame in a cell type-specific manner. J Virol.

[36] Liu X, Cohen JI (2014). Inhibition of Bim enhances replication of varicella-zoster virus and delays plaque formation in virus-infected cells. J Virol.

[37] Zhou G, Galvan V, Campadelli-Fiume G, Roizman B (2000). Glycoprotein D or J delivered in trans blocks apoptosis in SK-N-SH cells induced by a herpes simplex virus 1 mutant lacking intact genes expressing both glycoproteins. Journal of virology.

[38] Liu C, Vojnovic D, Kochevar IE, Jurkunas UV (2016). UV-A Irradiation Activates Nrf2-Regulated Antioxidant Defense and Induces p53/Caspase3-Dependent Apoptosis in Corneal Endothelial. Cells. Investigative ophthalmology & visual science.

[39] Lu X, Li C, Wang YK, Jiang K, Gai XD (2014). Sorbitol induces apoptosis of human colorectal cancer cells via p38 MAPK signal transduction. Oncology letters.

[40] Ding X, Wang D, Li L, Ma H (2016). Dehydroepiandrosterone ameliorates H_2_O_2_-induced Leydig cells oxidation damage and apoptosis through inhibition of ROS production and activation of PI3K/Akt pathways. Int J Biochem Cell Biol.

[41] Deruelle MJ, De Corte N, Englebienne J, Nauwynck HJ, Favoreel HW (2010). Pseudorabies virus US3-mediated inhibition of apoptosis does not affect infectious virus production. J Gen Virol.

[42] Qi ZL (2016). Salidroside protects PC12 cells from H2O2-induced apoptosis via suppressing NOX2-ROS-MAPKs signaling pathway. Nan fang yi ke da xue xue bao = Journal of Southern Medical University.

[43] Liu, X. R. *et al*. Propofol attenuates H_2_O_2_-induced oxidative stress and apoptosis via the mitochondria- and ER-medicated pathways in neonatal rat cardiomyocytes. *Apoptosis: an international journal on programmed cell death*, 10.1007/s10495-017-1349-3 (2017). 10.1007/s10495-017-1349-3 28176145

[44] Shimizu Y, Miyakura R, Otsuka Y (2015). Nuclear receptor subfamily 4, group A, member 1 inhibits extrinsic apoptosis and reduces caspase-8 activity in H_2_O_2_-induced human HUC-F2 fibroblasts. Redox Rep.

[45] Cao J (2016). The 2A2 protein of Duck hepatitis A virus type 1 induces apoptosis in primary cell culture. Virus Genes.

[46] Logue SE, Martin SJ (2008). Caspase activation cascades in apoptosis. Biochemical Society transactions.

[47] Lalaoui N, Lindqvist LM, Sandow JJ, Ekert PG (2015). The molecular relationships between apoptosis, autophagy and necroptosis. Semin Cell Dev Biol.

[48] Jorgensen I, Rayamajhi M, Miao EA (2017). Programmed cell death as a defence against infection. Nat Rev Immunol.

[49] Vanden Oever MJ, Han JY (2010). Caspase 9 is essential for herpes simplex virus type 2-induced apoptosis in T cells. J Virol.

[50] Jerome KR (1999). Herpes simplex virus inhibits apoptosis through the action of two genes, Us5 and Us3. J Virol.

[51] Aubert M, Krantz EM, Jerome KR (2006). Herpes simplex virus genes Us3, Us5, and Us12 differentially regulate cytotoxic T lymphocyte-induced cytotoxicity. Viral Immunol.

[52] Chen S (2018). Duck stimulator of interferon genes plays an important role in host anti-duck plague virus infection through an IFN-dependent signalling pathway. Cytokine.

[53] Wu Y (2011). Establishment of real-time quantitative reverse transcription polymerase chain reaction assay for transcriptional analysis of duck enteritis virus UL55 gene. Virol J.

[54] Li L (2011). Expression and characterization of duck enteritis virus gI gene. Virol J.

[55] Loret S, Guay G, Lippe R (2008). Comprehensive characterization of extracellular herpes simplex virus type 1 virions. J Virol.

[56] Leroy, B., Gillet, L., Vanderplasschen, A. & Wattiez, R. Structural Proteomics of Herpesviruses. *Viruses* **8**, 10.3390/v8020050 (2016). 10.3390/v8020050 PMC4776205 26907323

[57] Ishihama Y (2005). Exponentially modified protein abundance index (emPAI) for estimation of absolute protein amount in proteomics by the number of sequenced peptides per protein. Mol Cell Proteomics.

